# Differences in perceived return-to-work barriers between manual and nonmanual workers

**DOI:** 10.1093/joccuh/uiaf075

**Published:** 2025-12-24

**Authors:** Shunsuke Inoue, Seiichiro Tateishi, Arisa Harada, Etsuko Hosoda, Masako Nagata, Koji Mori

**Affiliations:** Department of Occupational Health Practice and Management, Institute of Industrial Ecological Sciences, University of Occupational and Environmental Health, Kitakyushu, Japan; Disaster Occupational Health Center, University of Occupational and Environmental Health, Kitakyushu, Japan; Disaster Occupational Health Center, University of Occupational and Environmental Health, Kitakyushu, Japan; Department of Occupational Medicine, School of Medicine, University of Occupational and Environmental Health, Kitakyushu, Japan; Treatment and Occupational Life Support Center, Hospital of the University of Occupational and Environmental Health, Kitakyushu, Japan; Department of Occupational Medicine, School of Medicine, University of Occupational and Environmental Health, Kitakyushu, Japan; Department of Occupational Health Practice and Management, Institute of Industrial Ecological Sciences, University of Occupational and Environmental Health, Kitakyushu, Japan

**Keywords:** return to work, manual workers, chronic health conditions, workplace support, psychosocial factors, support for work-treatment balance

## Abstract

**Background:**

Return-to-work (RTW) support has become a growing priority in occupational health. Manual workers—who constitute over half of the global labor force—may face greater RTW barriers due to the physically demanding nature of their jobs. However, few studies have quantitatively compared the perceived RTW barriers between manual and nonmanual workers. This study aimed to compare perceived RTW barriers between manual and nonmanual workers with chronic conditions to inform the development of tailored support strategies.

**Methods:**

We analyzed 219 employed adults, either actively working or on certified leave, who attended consultations at one particular hospital between September 2019 and June 2020 to obtain support for balancing work and medical treatment. Perceived RTW barriers were assessed with a validated 10-category yes/no structured checklist (personal: work ability/psychological/health literacy; workplace: structure/system/support; intersectoral/social). Logistic regression was performed to compare barriers between manual and nonmanual workers.

**Results:**

Manual workers were significantly more likely to report barriers related to psychological impacts (odds ratio [OR] = 2.34) and workplace systems (OR = 2.88). Although work ability did not differ significantly by job type, it was the most frequently reported RTW barrier in both groups.

**Conclusions:**

Manual workers’ RTW challenges are characterized by psychological and organizational barriers. RTW programs should assess psychological readiness before resumption of duties and provide managerial training to address anxiety and loss of confidence, while implementing job-specific accommodations such as phased tasks, ergonomic adjustments, and light duties in coordination with health care providers.

## Teaser

**Key points**

**What is already known on this topic**

Workers with chronic conditions often face multiple barriers when returning to work (RTW). Among them, engagement in physically demanding work—that is, being a manual worker—has been identified as a factor that makes RTW more difficult. However, few studies have quantitatively examined how engagement in manual labor influences the perception of RTW barriers.

**What this study adds**

This study demonstrates that manual workers are significantly more likely than nonmanual workers to report RTW barriers related to psychological impacts and workplace systems. Although work ability was the most frequently reported barrier across both groups, it did not differ significantly by job type.

**How this study might affect research, practice, or policy**

This study emphasizes the need for occupation-specific RTW systems that address psychological and organizational barriers among manual workers. In practice, assessing psychological readiness before resumption of duties and training supervisors to support workers with anxiety or loss of confidence are essential. Coordinated efforts between health care providers and employers can facilitate effective, job-specific accommodations and promote sustainable RTW in physically demanding sectors.

## 1. Introduction

Recent advances in medical care have enabled many people with chronic conditions to remain in the workforce, and roughly one-quarter to one-third of workers in industrialized countries require ongoing treatment.^1,2^ Beyond the burden on the individual, prolonged sick leave and job loss create staffing pressures for employers,^3^ making effective return-to-work (RTW) support an occupational health priority.

Previous studies have shown that barriers to RTW among workers with chronic conditions are influenced by both workplace factors (eg, job demands, social support) and individual factors (eg, age, sex, psychological status).^4,5^ In the Japanese context, where intersectoral collaboration among health care providers, workplaces, and families is emphasized,^6^ workplace climate also shapes psychosocial barriers; for example, higher perceived organizational justice is associated with greater illness reporting among workers with chronic diseases.^7^ At the organizational level, receipt of workplace accommodations differs by disease, suggesting selective rather than routine implementation of adjustments.^8^ Together, these observations highlight the need to examine which psychosocial and organizational barriers are most salient in this setting.

Among workers with chronic conditions, those engaged in physically demanding occupations may face significant challenges in maintaining or resuming employment. According to a report by the International Labour Organization, over half of the global workforce—approximately 1.2 billion individuals—are engaged in manual labor.^9^ Manual labor is defined as work requiring physical effort such as standing, lifting, pushing, or pulling heavy objects.^10^ Manual workers are estimated to be approximately 3 times more likely than nonmanual workers to develop musculoskeletal disorders^11^ and are also at greater risk of prolonged sick leave.^12,13^ Recent research has suggested that RTW is especially difficult for manual workers with various chronic conditions, including cancer, cerebrovascular disease, cardiovascular disease, and musculoskeletal disorders.^4,14–18^ In other words, engaging in manual labor itself is widely recognized as a potential barrier to successful RTW.

However, although manual work is often cited as a barrier to RTW, little is known about which barriers manual workers themselves more frequently perceive compared with nonmanual workers. Using a validated 10-category checklist, we examined the domain-specific pattern of perceived barriers—particularly psychological and organizational domains—rather than treating manual work as a single occupational label. Clarifying these subjective difficulties can guide targeted accommodations and clinician–employer coordination, aiming to improve RTW and reduce recurrent sick leave. Therefore, we quantified domain-specific differences in perceived RTW barriers between manual and nonmanual workers with chronic conditions in Japan. We hypothesized that manual workers would show higher odds of endorsing psychological and organizational barriers.

## 2. Methods

### 2.1. Study period and participants

This study included patients who visited a single university hospital between September 1, 2019, and June 30, 2020, seeking support for balancing work and medical treatment. Participant eligibility was determined during hospitalization briefings, informed consent sessions for treatment initiation, or upon referral by the attending physician. Individuals were included in the analysis only if they held an active employment contract—either currently working or on certified medical leave. Those whose employment had already been terminated at the time of consultation were excluded from the study.

### 2.2. Measurement of RTW barriers

We assessed perceived RTW barriers with a 10-category checklist originally developed from semistructured interviews across 3 disease groups (stroke, heart disease, cancer) and analyzed using qualitative content analysis with systematic text condensation, following COREQ/SRQR guidance.^6^ The development generated 3 higher-order themes and 10 sub-themes (work ability, psychological impacts, health literacy, social status, family background, workplace structure, workplace system, workplace support, intersectoral collaboration, social resources). Content/face validity were established during development through iterative coding, expert review, and a priori agreement thresholds for category decisions (trustworthiness predefined as ≥70% inter-rater agreement among 3 researchers, adding a fourth if <70% and repeating until ≥70% was achieved). In the present study, trained interviewers recorded each category as yes/no after a standardized explanation. The checklist has since been applied in clinical settings beyond the original qualitative sample and made publicly available by Japan’s Ministry of Health, Labour and Welfare.^19–21^

### 2.3. Data collection

Face-to-face interviews were conducted by certified coordinators for balancing work and medical treatment (eg, physicians, nurses, medical social workers, or licensed public psychologists) in outpatient clinics or inpatient consultation rooms at the hospital. Each interview, lasting approximately 30-60 minutes, was conducted by 1 or 2 certified interviewers. Basic demographic and clinical information (sex, age, and primary diagnosis) was extracted from medical records. Diagnoses were classified according to the International Classification of Diseases, 10th Revision.

Job tasks were evaluated using the following 11 items developed based on job descriptions from O*NET (Occupational Information Network), which have also been adopted in a report by the Ministry of Health, Labour and Welfare of Japan^19^: clerical work, computer work, multitasking, fine motor tasks using fingers, tasks requiring attention to detail, physically active tasks, physically demanding work, tasks involving interpersonal communication, work involving dust or hazardous substances, work in hot or cold environments, and tasks involving personal or environmental danger such as working at heights or driving. Participants selected all applicable tasks in a multiple-response format. Even if participants engaged in multiple duties, they were classified as manual workers if they reported performing either “physically active tasks” or “physically demanding work.” Conversely, participants who did not report either “physically active tasks” or “physically demanding work” were classified as nonmanual workers, even if they reported environmental risks such as “dust/hazardous work” or “work at heights.”

To address potential confounding by employment arrangements, we additionally adjusted for employment type (civil servant, permanent employee, part-time, contract/temporary) and company size (<50, 50-299, ≥300 employees). Both variables were entered as categorical indicators.

### 2.4. Statistical analysis

Associations between engagement in manual labor and each of the 10 RTW barrier categories were analyzed using logistic regression. Odds ratios (ORs) were estimated using 4 models with progressively adjusted covariates:

- Model 1: unadjusted (manual labor only).
- Model 2: adjusted for sex and age.
- Model 3: additionally adjusted for disease classification.
- Model 4: additionally adjusted for employment type and company size.

Because several RTW barrier categories had relatively few positive responses, leading to events per variable (EPV) below conventional thresholds, we additionally ran Firth bias-reduced penalized logistic regression models for all 10 outcomes. These models used the same set of covariates as Model 4.

To facilitate interpretation of the magnitude of group differences, adjusted predicted probabilities for manual and nonmanual workers were calculated based on Model 4. Absolute differences were expressed in percentage points (pp) with 95% CIs.

Each RTW barrier was treated as a binary outcome (yes/no). Two-tailed *P* < .05 was considered statistically significant. Analyses were conducted using Stata Statistical Software, Release 19 (StataCorp LLC, College Station, TX, USA).

### 2.5. Ethical considerations

This study was approved by the Ethics Review Committee of the University of Occupational and Environmental Health, Japan (Approval No. R3-002). It was conducted in accordance with the Ethical Guidelines for Medical and Health Research Involving Human Subjects (February 2017 revision). Information on the opt-out procedure and data protection was made publicly available.

## 3. Results

Of the 261 patients who sought support for balancing work and medical treatment during the study period, 219 were included in the final analysis. Seven individuals were excluded due to unemployment, and 35 were excluded due to missing interview data. Participant characteristics are presented in Table 1. The final sample included 119 men (54%) and 100 women (46%), with a mean age of 48 years. Among them, 113 (52%) were classified as manual workers.

**Table 1:** Participants’ characteristics.

	**Manual workers (*n* = 113)**	**%**	**Nonmanual workers (*n* = 106)**	**%**
**Sex**				
**Men**	56	50	63	59
**Women**	57	50	43	41
**Age, y**				
**≤19**	0	0	1	1
**20-29**	11	10	4	4
**30-39**	16	14	13	12
**40-49**	30	27	30	28
**50-59**	34	30	36	34
**60-69**	21	19	21	20
**≥70**	1	1	1	1
**Disease classification**				
**Neoplasms**	42	37	47	44
**Diseases of the blood and blood-forming organs and certain disorders involving the immune mechanism**	10	9	4	4
**Endocrine, nutritional, and metabolic diseases**	5	4	6	6
**Mental and behavioral disorders**	2	2	8	8
**Diseases of the nervous system**	6	5	5	5
**Diseases of the eye, adnexa, ear, and mastoid process**	3	3	5	5
**Diseases of the circulatory system**	7	6	11	10
**Diseases of the respiratory system**	0	0	1	1
**Diseases of the digestive system**	6	5	4	4
**Diseases of the skin and subcutaneous tissue**	1	1	2	2
**Diseases of the musculoskeletal system and connective tissue**	20	18	9	8
**Diseases of the genitourinary system**	4	4	2	2
**Congenital malformations, deformations, and chromosomal abnormalities**	2	2	0	0
**Symptoms, signs, and abnormal clinical and laboratory findings, not elsewhere classified**	1	1	0	0
**Injury, poisoning, and certain other consequences of external causes**	4	4	2	2
**Categories of RTW barriers (multiple answers possible)**				
**Work ability**	78	69	60	57
**Psychological impacts**	66	58	48	45
**Health literacy**	31	27	22	21
**Social status**	32	28	26	25
**Family background**	15	13	13	12
**Workplace structure**	13	12	17	16
**Workplace system**	25	22	12	11
**Workplace support**	25	22	13	12
**Intersectoral collaboration**	12	11	11	10
**Social resources**	23	20	13	12

Abbreviation: RTW, return-to-work.

Regarding disease classification, the most common diagnosis was neoplasms (*n* = 89; 41%), followed by musculoskeletal and connective tissue disorders (*n* = 29; 13%), and circulatory system diseases (*n* = 18; 8%).

In total, participants reported 555 RTW barriers across the 10 categories: 320 by manual workers and 235 by nonmanual workers. The most frequently cited barrier in both groups was work ability, followed by psychological impacts and social status.

The results of the logistic regression analysis examining the associations between engagement in manual labor and each RTW barrier category are shown in Table 2. In Model 3 (adjusted for sex, age, and disease classification), manual work was associated with higher odds of endorsing psychological impacts (OR 1.98; 95% CI, 1.11-3.54; *P* = .022), workplace systems (OR 2.71; 95% CI, 1.16-6.32; *P* = .021), and workplace support (OR 2.49; 95% CI, 1.12-5.53; *P* = .025). These patterns were broadly consistent across simpler specifications: the workplace systems domain was already significant in Models 1-2, while psychological impacts and workplace support showed similar directions with *P* values approaching significance. Although work ability and social resources did not meet conventional significance thresholds, their ORs exceeded 1.5 in multiple models, suggesting possible signals worth further investigation. After additional adjustment for employment type and company size in Model 4, associations remained for psychological impacts (OR 2.34; 95% CI, 1.21-4.55; *P* = .012) and workplace systems (OR 2.88; 95% CI, 1.12-7.36; *P* = .027). Work ability was borderline (OR 2.00; 95% CI, 0.99-4.02; *P* = .050), and other domains were nonsignificant (eg, workplace support OR 1.78; 95% CI, 0.75-4.27; *P* = .193).

**Table 2:** Association between manual labor and RTW barriers.

**Categories of RTW Barriers**	**Model 1** ^**a**^			**Model 2** ^**b**^			**Model 3** ^**c**^			**Model 4** ^**d**^			**Model 5** ^**e**^		
	**OR**	**95% CI**	***P***	**OR**	**95% CI**	***P***	**OR**	**95% CI**	***P***	**OR**	**95% CI**	***P***	**Coef.**	**95% CI**	***P***
**Work ability**	1.71	0.98 to 2.97	.058	1.64	0.93 to 2.87	.085	1.62	0.89 to 2.95	.112	2.00	0.99 to 4.02	.050	0.635	−0.04 to 1.31	.063
**Psychological impacts**	1.7	0.99 to 2.90	.053	1.69	0.98 to 2.90	.059	1.98	1.11 to 3.54	.022	2.34	1.21 to 4.55	.012	0.772	0.13 to 1.42	.019
**Health literacy**	1.44	0.77 to 2.70	.250	1.40	0.74 to 2.65	.296	1.44	0.71 to 2.90	.309	1.40	0.96 to 2.97	.381	0.368	−0.36 to 1.09	.319
**Social status**	1.21	0.67 to 2.22	.526	1.27	0.69 to 2.35	.436	1.37	0.71 to 2.63	.351	1.46	0.65 to 3.31	.360	0.391	−0.38 to 1.16	.321
**Family background**	1.09	0.49 to 2.42	.823	1.13	0.50 to 2.53	.758	1.24	0.52 to 2.97	.62	0.94	0.35 to 2.52	.902	−0.538	−0.96 to 0.88	.936
**Workplace structure**	0.68	0.31 to 1.48	.331	0.69	0.31 to 1.50	.345	0.66	0.29 to 1.53	.336	0.56	0.22 to 1.46	.238	0.460	−1.46 to 0.39	.254
**Workplace system**	2.23	1.05 to 4.70	.036	2.20	1.04 to 4.70	.040	2.71	1.16 to 6.32	.021	2.88	1.12 to 7.36	.027	0.914	0.03 to 1.80	.042
**Workplace support**	2.03	0.98 to 4.22	.057	1.97	0.94 to 4.11	.073	2.49	1.12 to 5.53	.025	1.78	0.75 to 4.27	.193	−0.038	−0.38 to 1.30	.282
**Intersectoral collaboration**	1.03	0.43 to 2.44	.953	0.94	0.39 to 2.29	.895	1.07	0.43 to 2.67	.892	0.92	0.34 to 2.48	.867	0.100	−0.83 to 1.03	.833
**Social resources**	1.83	0.87 to 3.83	.110	1.85	0.88 to 3.89	.107	1.94	0.89 to 4.20	.094	1.99	0.82 to 4.88	.130	0.640	−0.24 to 1.52	.153

Abbreviations: Coef., coefficient; OR, odds ratio; RTW, return to work.

^a^Model 1: unadjusted.

^b^Model 2: adjusted for sex and age.

^c^Model 3: additionally adjusted for disease classification.

^d^Model 4: additionally adjusted for employment type and company size.

^e^Model 5: Firth-penalized logistic regression using the same covariates as Model 4.

Sample size adequacy was assessed using the EPV criterion, as proposed by Peduzzi et al.^22^ In Models 1 and 2, most predictors exceeded the threshold of EPV ≥10. In Model 3, five variables fell below this threshold, with intersectoral collaboration showing the lowest EPV (5.75). These findings require cautious interpretation, although workplace system remained significant across all models.

When we applied the Firth bias-reduced logistic regression to account for sparse outcomes (Model 5), the direction and magnitude of the associations were largely unchanged. Manual work remained associated with psychological impacts and workplace systems, supporting the robustness of the main findings.

Table 3 presents the adjusted predicted probabilities of endorsing each RTW barrier for manual and nonmanual workers based on Model 4. In particular, manual workers showed higher predicted probabilities for psychological impacts (0.64 vs 0.45; +19.2 pp; 95% CI, 4.6-33.7) and workplace system (0.23 vs 0.10; +12.8 pp; 95% CI, 1.9-23.6).

**Table 3:** Predicted probabilities of RTW barriers by work type.

**Categories of RTW barriers**	**Nonmanual (95% CI)**	**Manual (95% CI)**	**Absolute difference, pp (95% CI)**	***P* value**
**Work ability**	0.55 (0.45 to 0.66)	0.70 (0.61 to 0.80)	+15.1 (0.23 to 29.9)	.047
**Psychological impacts**	0.45 (0.34 to 0.55)	0.64 (0.54 to 0.74)	+19.2 (4.6 to 33.7)	.010
**Health literacy**	0.24 (0.15 to 0.33)	0.30 (0.21 to 0.39)	+5.9 (−7.2 to 18.9)	.380
**Social status**	0.22 (0.14 to 0.30)	0.28 (0.19 to 0.37)	+5.9 (−6.7 to 18.6)	.359
**Family background**	0.15 (0.07 to 0.23)	0.15 (0.07 to 0.22)	−0.7 (−12.3 to 10.9)	.902
**Workplace structure**	0.19 (0.10 to 0.28)	0.12 (0.05 to 0.19)	−7.2 (−18.9 to 4.6)	.233
**Workplace system**	0.10 (0.04 to 0.16)	0.23 (0.14 to 0.32)	+12.8 (1.9 to 23.6)	.021
**Workplace support**	0.14 (0.07 to 0.22)	0.23 (0.14 to 0.31)	+8.1 (−4.0 to 20.2)	.188
**Intersectoral collaboration**	0.12 (0.05 to 0.19)	0.12 (0.05 to 0.18)	−0.9 (−11.0 to 9.3)	.867
**Social resources**	0.12 (0.05 to 0.19)	0.21 (0.12 to 0.29)	+8.8 (−2.4 to 19.9)	.124

Abbreviations: pp, percentage points; RTW, return to work.

## 4. Discussion

This study revealed that manual workers with chronic conditions were significantly more likely than nonmanual workers to perceive RTW barriers related to psychological impacts and workplace systems. Barriers related to psychological impacts refer to issues such as diminished confidence in performing job tasks, anxiety about symptom recurrence or ongoing treatment, and emotional distress—challenges that are common among workers attempting to resume employment following chronic conditions.^23–25^ van der Kemp et al^23^ demonstrated that depressive symptoms and reduced self-efficacy hinder RTW among workers recovering from mild to moderate stroke, underscoring psychological factors as a critical barrier to RTW. Importantly, engaging in manual labor itself may amplify psychological barriers. Marom et al^24^ reported that manual workers with hand injuries experienced lower self-efficacy and trauma-related symptoms, both of which were significantly associated with delayed RTW. These workers often face uncertainty about their ability to resume the same physically demanding tasks, potentially exacerbating psychological distress. Furthermore, Utzet et al^25^ identified that manual workers are chronically exposed to high workloads and limited job control—factors closely linked to poor mental health outcomes. These findings underscore how the physical demands and job conditions inherent in manual labor can profoundly impact psychological well-being. Taken together, these results suggest that, for manual workers, psychological barriers to RTW are shaped not only by physical recovery but also by internal concerns, including doubts about their ability to resume previous tasks or fulfill workplace expectations. The present study’s findings are consistent with previous research and suggest that manual workers tend to face greater psychological risks, which may contribute to difficulties in achieving successful RTW.

Workplace systems barriers denote insufficient or poorly implemented RTW support at the organizational level. Although such issues may affect workers across job types,^26,27^ manual workers are particularly vulnerable to gaps in execution, even where formal systems exist.^8,27–29^ Harada et al^8^ reported that workers with musculoskeletal conditions receive less workplace support, suggesting inequitable application of RTW measures. Beurden et al^28^ noted that physically demanding temporary jobs often lack flexible accommodations and impose administrative burdens, limiting policy effectiveness. Williams-Whitt^29^ similarly found that inconsistent managerial practices and perceived burdens of accommodation further reduce the effectiveness of RTW systems, especially for physically intensive and repetitive jobs. Our findings align with this literature, showing that manual workers perceive workplace systems as RTW barriers more often than nonmanual workers. This pattern may indicate that physically demanding roles are perceived as less adaptable to existing RTW systems, as job modification is often constrained by task requirements and safety considerations. Similar to the differing perceptions reported by Grunfeld et al^30^ between cancer survivors and their employers, such findings may reflect perceptual or organizational gaps in how RTW policies are experienced across job types, rather than a direct causal effect of job type itself. These results underscore that the mere existence of policies is insufficient; RTW support must incorporate mechanisms to ensure equitable and practical implementation, especially in physically demanding occupations.

Barriers related to workplace support—insufficient assistance from supervisors or colleagues—are widely recognized as obstacles to RTW.^25,30–33^ In this study, manual workers were more likely than nonmanual workers to report a lack of support as a barrier. However, this association was significant only in the partially adjusted model (Model 3) and disappeared after adjusting for employment type and company size, suggesting that organizational factors may partly explain the difference. A recent nationwide study also found that perceived organizational support tends to be lower in medium-sized firms and varies by supervisory support.^34^ These findings together indicate that workplace support barriers may reflect not only job characteristics but also organizational context.

Although “work ability” did not differ significantly between manual and nonmanual workers, it was the most frequently reported RTW barrier in both groups. Upon returning to work, manual workers may face expectations to endure continuous physical demands, whereas nonmanual workers are expected to maintain sustained attention and cognitive processing. These differing job-specific expectations may lead workers to perceive their inability to meet these standards as a major barrier to RTW. In the Japanese context, previous literature has noted that cultural constructs such as amae and oime can heighten sensitivity to role fulfillment and to others’ evaluations.^35^ Although our study did not directly measure these cultural factors, they may offer a contextual, hypothesis-generating lens to interpret why some workers experience inability to meet expected roles as a psychological burden.

Our findings highlight the need for more effective RTW systems addressing the psychological and organizational barriers disproportionately experienced by manual workers. Rather than applying uniform policies, workplaces should develop flexible, job-specific RTW strategies—particularly for physically demanding roles—by implementing phased tasks, ergonomic adjustments, and temporary light-duty assignments. Assessing workers’ psychological readiness before resumption of duties and providing managerial training to help supervisors address anxiety and loss of confidence are essential, along with coordination between health care providers and employers to ensure consistent implementation. In clinical settings, it is also important to assess psychological readiness and occupation-related challenges to achieve sustainable RTW.

This study has several limitations. It was conducted at a single institution with a high proportion of cancer patients, which may have introduced sampling bias and limits the generalizability of the findings to other settings, industries, and employment systems. Multi-center, multi-year studies are therefore needed. RTW outcomes were not assessed, so the direct impact of the identified barriers remains unclear. Job tenure was not collected, which may leave residual confounding. RTW barriers were assessed through face-to-face structured interviews using standardized materials; however, interviewer effects and social desirability (response) bias cannot be fully excluded, and these factors may have led to over- or under-reporting of some barrier categories. In addition, several barrier categories had few positive responses, leading to EPV values below 10 (eg, intersectoral collaboration had EPV = 5.75 in Model 3), which may limit the stability of the estimates. To address sparse data, we additionally ran a Firth penalized logistic regression and confirmed broadly similar directions of association, but these categories should still be interpreted with caution. Only workers who visited the medical institution to seek support for balancing work and medical treatment were included. Individuals who did not perceive a need for support or who had abandoned the idea of returning to work due to severe barriers were not represented. As such, there is a possibility of selection bias, and the findings may not be generalizable to all workers. Furthermore, job classification was based on participants’ self-reported job tasks. Although this approach is commonly used in occupational health research, it may not fully capture the objective nature of their work. As a result, some degree of misclassification bias cannot be ruled out, particularly when job roles include both manual and nonmanual elements or when participants subjectively interpret what constitutes “physical” work. These limitations should be taken into account when translating the findings into practice.

## 5. Conclusions

This study revealed that manual workers with chronic conditions were more likely than nonmanual workers to perceive RTW barriers related to psychological impacts and workplace systems. These findings underscore the need for RTW systems that address both psychological and organizational factors disproportionately affecting manual workers. In practice, assessing psychological readiness before resumption of duties and providing managerial training to help supervisors address anxiety and loss of confidence are essential for promoting sustainable RTW. Flexible, job-specific accommodations—such as phased tasks, ergonomic adjustments, and temporary light-duty assignments—should be implemented, particularly in physically demanding sectors. Coordinated efforts between health care providers and employers can facilitate consistent and equitable support, ensuring that RTW programs are both feasible and effective across diverse occupational contexts.

## Data Availability

The data underlying this article are available from the corresponding author upon reasonable request.
